# A multi-component model of the dynamics of salt-induced hypertension in Dahl-S rats

**DOI:** 10.1186/1472-6793-9-20

**Published:** 2009-10-29

**Authors:** Violeta I McLoone, John V Ringwood, Bruce N Van Vliet

**Affiliations:** 1 Department of Electronic Engineering, National University of Ireland Maynooth, Maynooth, Co. Kildare, Ireland; 2 Division of BioMedical Sciences, Faculty of Medicine, Memorial University of Newfoundland, St. John's, Newfoundland A1B 3V6, Canada

## Abstract

**Background:**

In humans, salt intake has been suggested to influence blood pressure (BP) on a wide range of time scales ranging from several hours or days to many months or years. Detailed time course data collected in the Dahl salt-sensitive rat strain suggest that the development of salt-induced hypertension may consist of several distinct phases or components that differ in their timing and reversibility. To better understand these components, the present study sought to model the dynamics of salt-induced hypertension in the Dahl salt sensitive (Dahl-S) rat using 3 sets of time course data.

**Results:**

The first component of the model ("Acute-Reversible") consisted of a linear transfer function to account for the rapid and reversible effects of salt on BP (ie. acute salt sensitivity, corresponding with a depressed slope of the chronic pressure natriuresis relationship). For the second component ("Progressive-Irreversible"), an integrator function was used to represent the relatively slow, progressive, and irreversible effect of high salt intake on BP (corresponding with a progressive salt-induced shift of the chronic pressure natriuresis relationship to higher BP levels). A third component ("Progressive-Reversible") consisted of an effect of high salt intake to progressively increase the acute salt-sensitivity of BP (ie. reduce the slope of the chronic pressure natriuresis relationship), amounting to a slow and progressive, yet reversible, component of salt-induced hypertension. While the 3 component model was limited in its ability to follow the BP response to rapid and/or brief transitions in salt intake, it was able to accurately follow the slower steady state components of salt-induced BP changes. This model exhibited low values of mean absolute error (1.92 ± 0.23, 2.13 ± 0.37, 2.03 ± 0.3 mmHg for data sets 1 - 3), and its overall performance was significantly improved over that of an initial model having only 2 components. The 3 component model performed well when applied to data from hybrids of Dahl salt sensitive and Dahl salt resistant rats in which salt sensitivity varied greatly in its extent and character (mean absolute error = 1.11 ± 0.08 mmHg).

**Conclusion:**

Our results suggest that the slow process of development of salt-induced hypertension in Dahl-S rats over a period of many weeks can be well represented by a combination of three components that differ in their timing, reversibility, and their associated effect on the chronic pressure natriuresis relationship. These components are important to distinguish since each may represent a unique set of underlying mechanisms of salt-induced hypertension.

## Background

A high level of dietary salt or sodium intake is thought to contribute importantly to the etiology [1] and prevalence [2] of hypertension. The physiological basis of the link between salt and blood pressure (BP) has been well investigated, resulting in the identification of a number of factors and mechanisms that contribute to the salt-sensitivity of BP [3-5]. The focus of the present study concerns the detailed time course of the effects of salt loading on BP, and what this may tell us about the component structure or processes underlying the development of salt-induced hypertension.

At one extreme, salt loading and restriction have been widely described to affect BP within several days or weeks, and changes on this time scale have often been used to categorize individuals as salt-sensitive or salt-resistant [6,7]. This acute form of BP salt-sensitivity corresponds with an altered slope of the steady state relationship between salt intake and BP (the chronic pressure-natriuresis relationship) [3,8], and has been a focus of much research concerning the mechanisms and features of salt-induced hypertension. At the other extreme, high dietary salt intakes have also been demonstrated to affect BP on a much slower time scale of many months or years [9-12]. In some studies, these slow and progressive effects of salt have been found to be partly irreversible [13-15], the irreversible characteristic corresponding with a shift of the chronic pressure natriuresis relationship to higher BP levels [13,14]. Finally, an effect of sustained salt loading to alter the slope of the chronic pressure natriuresis relationship, amounting to a salt induced worsening of acute salt-sensitivity, has also been suggested [13,14]. Thus, the impact of high dietary salt intake on BP may represent a combination of processes that differ with respect to their timing, reversibility, and specific effect on the chronic pressure natriuresis relationship.

The Dahl rat (Dahl-S) strain is a widely investigated experimental model of genetic salt-sensitivity in which the time course of salt-induced hypertension has been studied in detail. In a previous study of this strain, we found that the characteristics of both acute and slow-progressive forms of salt-induced hypertension appeared to coexist [13]. In the present study, we used data from Dahl-S rats to investigate whether the time course of salt-induced hypertension in this strain could be well represented as a multi-component process. To extend previous theoretical analyses of the acute salt sensitivity of BP which have focused on the chronic pressure natriuresis relationship [3,8], each component was designed to correspond with a defining feature (e.g. slope or position) of the chronic pressure natriuresis relationship. Constructed in this manner, the model was able to describe not only the progression of salt-induced hypertension, but also the corresponding progressive alterations of the chronic pressure natriuresis relationship.

## Methods

### Data sets

The Dahl salt sensitive rat represents an experimental model in which the time course of salt-induced hypertension has been documented in greatest detail [13]. We developed our model using all detailed time course data available to us for this strain. This consisted of two previously published sets and one previously unpublished set of data that illustrate the time course of BP responses to salt loading in Dahl salt sensitive rats. A fourth set of previously published time course data obtained from hybrids of Dahl-S and Dahl-R rats was subsequently used to investigate whether the model was generally robust. All experiments were approved by Memorial University's Institutional Animal Care Committee.

**Figure 1:**
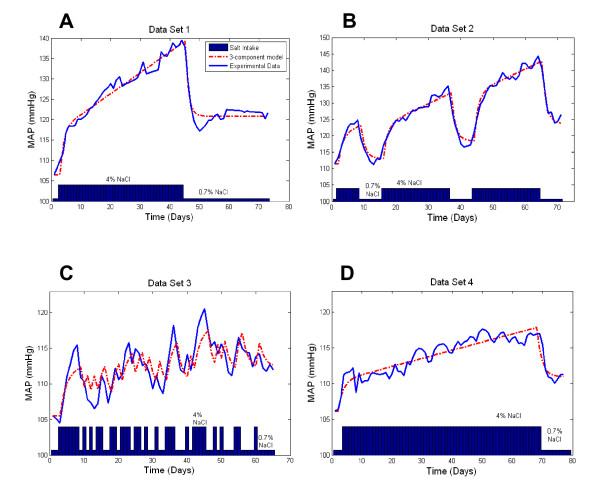
**Comparison of model responses with the original data**. Model output is compared with the group-average mean arterial pressure (MAP) data for each of the four data sets (A - D). The time course of the change in dietary salt between low (0.7% NaCl) and high (4% NaCl) levels is shown in blue at the base of each graph.

*Data set 1 *(Figure 1A) consisted of previously published [13] time course data of BP changes for 9 male Dahl-S rats exposed to a step increase in salt intake (1 control week of regular (0.7%) salt diet, followed by 6 weeks of high (4%) salt diet, followed by a 4 week recovery period on regular salt diet).

*Data set 2 *(Figure 1B) provides the previously published [13] time course of BP changes for 5 male Dahl-S rats during a sequence of step increases in salt intake which had been used to explore changes in an irreversible component of salt induced BP changes.

**Figure 2:**
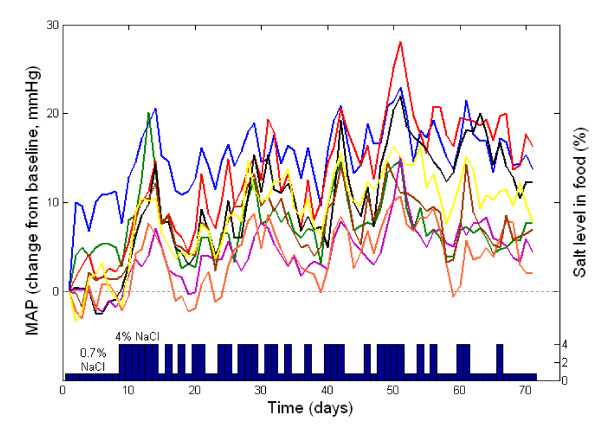
**Illustration of the original blood pressure data of data set 3**. The plot shows the salt-induced change in mean arterial pressure (MAP) of the 8 individual Dahl-S rats in data set 3. The average response of the 8 animals is shown in Figure 1C. The time course of the change in dietary salt between low (0.7%) and high (4%) levels is shown in blue at the base of the graph. The upward trend in MAP over the course of the intermittent salt exposure was significant, amounting to 9.6 ± 1.9 mmHg (P < 0.01).

*Data set 3 *is a previously unpublished data set documenting the BP response of Dahl-S rats (males, Brookhaven Strain, ~3 months of age) to a sequential switching of dietary salt between regular (0.7%) and high (4%) levels according to a pseudo-random binary sequence. Figure 1C illustrates the mean BP response of the 8 individual Dahl-S rats used in this protocol, while the response of individual animals is provided in Figure 2. This previously unpublished data set was originally produced by us in the course of a pilot study which sought to use linear transfer function analysis to contrast differences in the rapid and slow components of salt-induced hypertension in this model. This analysis was not pursued because standard linear analysis methods were realized to be inappropriate when some of the components of the salt-induced BP response were found to be nonlinear (ie. partly irreversible: by the end of the protocol for this data set, BP levels on low salt diet were significantly elevated above that of the baseline period, P < 0.05. An irreversible nature of salt induced hypertension in this model is even more evident in other data sets, Figure 1). Being available, this detailed data set was therefore included in model development and evaluation.

To record BP for data set 3, a BP telemeter (Ta11Pa-C40, Data Sciences International) was surgically implanted in each rat at 10 - 11 weeks of age under pentobarbital (55 mg/kg i.p.) or ketamine-xylazine (90 and 5-10 mg/kg i.m., respectively) anesthesia, as previously described [16]. Penicillin G (30,000 U/kg, i.m.) was administered at the time of surgery. Following a recovery period of at least 11 days, BP was recorded continuously for 72 days, each daily mean BP level being calculated as the average level of mean arterial pressure sampled once each minute, 1440 times per day. During this recording period, the dietary salt level was manipulated on a daily basis in the following pseudo-random binary sequence, with each "0" indicating a 24 h exposure to a regular salt (0.7% NaCl) diet and "1" indicating a 24 h exposure to a high salt (4% NaCl) diet): 0000000001 1111101010 1100110111 0110100100 1110001011 1100101000 110000100000 (see Figure 1C and Figure 2). To maintain the effects of occasional disturbance constant throughout the protocol, cages were changed on a daily basis between 11 am and 12 noon, and any changes to the diet were also made at that time. Telemetry records were inspected on a daily basis in order that occasional artifacts could be detected and excluded from analysis. The BP level was calculated as the mean of all BP samples recorded between 12 noon on one day and the next. At the end of the protocol, rats were killed by anesthetic overdose and the telemeters were removed and cleaned. Telemeters were calibrated on several occasions before and following the protocol, and the change in offset with time was calculated by linear regression. In 11 cases in which the regression was statistically significant (P < 0.05), we used the regression data to correct offset drift on a daily basis, with daily increments in offset correction ranging from 0.004 to 0.09 mmHg over the course of the protocol. In 2 cases in which the regression was not significant, a single mean offset correction value was uniformly applied to all data.

Data set 4 (Figure 1D) consisted of the previously published [13] time course of the BP response to a step increase in dietary salt among 13 male hybrid rats (F_2 _progeny of a cross between Dahl-S and Dahl-R rats) whose salt-sensitivity ranges between those of the Dahl-S and Dahl-R strains. The experimental protocol consisted of a control week of regular (0.7%) salt diet, followed by high (4%) salt diet for 10 weeks, followed by a return to regular salt diet for 1 week.

### Numerical methods

During development of the model structure, we used mean values for each data set as inputs to the model. Subsequently, models were parameterised for each individual animal and errors were estimated. MatLab's Simulink (The Mathworks Inc, Natick, MA, USA) modelling environment was used for model implementation. The numerical optimization was done using Neleder-Mead simplex algorithm [17] implemented in the "fminsearch" function in the MatLab Optimisation Toolbox. The objective function to be minimised (*J*_*k*_) was defined as the Mean Squared Error (MSE) between model results and experimental data:

• y_i _is the actual experimental BP data, and

• ŷ_i _is the BP response obtained from the model simulations.

Error results are presented in the form of the mean absolute error (MAE), which indicates the average difference between each point in the data and the corresponding point in the model results in mmHg. During model development, model performance was evaluated by visual inspection of the dynamic performance of the model output relative to the original data sets and by comparison of each model's MAE values. An F-test was subsequently used to evaluate improvements in the performance of the model (residual sum of squares) relative to changes in model complexity (degrees of freedom) [18].

## Results

### Model performance for data from Dahl-S rats (data sets 1-3)

**Figure 3:**
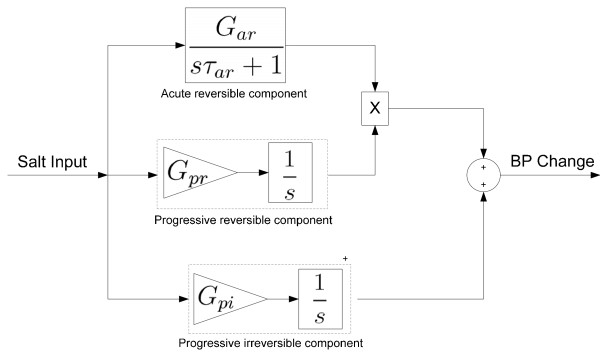
**Illustration of the structure of the 3 component model**. Each of the individual three component parts of the model are outlined and labelled. G_*AR*_, gain of the Acute-Reversible component; τ_*AR*_, time constant of the Acute-Reversible component; G_*PR*_, gain of the Progressive-Reversible component; G_*PI*_, gain of the Progressive-Irreversible component.

The first component ("Acute-Reversible", AR) of the model consisted of a first order transfer function with time constant τ_AR _and steady state gain G_AR _(Figure 3). This component was selected to represent the dynamics of the well described reversible change in BP that occurs over the days to weeks following a step change in salt intake in salt sensitive subjects (e.g. humans [19], dogs [20], mice [21], rats [22], monkeys [23]), with the value G_AR _serving to scale the degree of acute salt sensitivity of BP (ie. G_AR _= change in steady state BP level/change in dietary salt content above the baseline level of 0.7% NaCl) and being inversely proportional to the slope of the chronic pressure natriuresis relationship. The second component ("Progressive-Irreversible", PI) consisted of a simple integrator with gain G_PI _(Figure 3). This component was selected to represent the slow, progressive, and irreversible progression of salt-induced hypertension apparent in Dahl-S rats [13] (but also evident on a slower time scale in regular outbred rats [9,15,24]), and corresponds with an effect of chronic high salt intake to shift the chronic pressure natriuresis relationship to higher BP levels. The value G_PI _scales the sensitivity of this shift to salt intake, the shift in BP amounting to the product G_PI_·(increase in dietary salt content above baseline of 0.7% NaCl)·(time spent above baseline salt level, in days). A third component ("Progressive-Reversible", PR) consisted of a simple integrator term with gain G_PR _that imposed a progressive and irreversible increase in the effect of the gain G_AR _of the 1^st ^component (Acute-Reversible) of the model in proportion to the extent and duration of exposure to high salt (Figure 3). G_PR _serves to scale this salt dependent increase in acute salt sensitivity, effectively increasing the effect of G_AR _by the product G_PR_·(increase in dietary salt content above baseline level of 0.7% NaCl)·(time spent above baseline salt level, in days). Like the Progressive-Irreversible component, this 3^rd ^component provided the 3 component model with a slow and progressive BP response to a sustained increase in salt intake. However, in contrast with the Progressive-Irreversible component, this 3rd component represents a progressive effect of high salt intake to increase acute salt-sensitivity (ie. to progressively reduce the slope of the CPNR). This component was intended to correspond to the salt-induced worsening of acute salt sensitivity that is apparent in the progressively increasing BP response to salt restriction in data set 2 [13]. Such a process is also consistent with the slow and progressive but reversible form of salt-induced rise in BP evident in time course data from chimpanzees [10].

The 3 component model was able to accurately follow BP responses to relatively sustained changes in salt intake, but was limited in its ability to reproduce the most rapid BP transients associated with sudden and/or brief transitions in salt intake (particularly in the case of data set 3, obtained from an experimental protocol featuring frequent and brief changes in salt intake). Mean error values for the 3 component model were generally low (<3 mmHg, Table 1). An F-test indicated that, for data sets 1 and 2, model performance was statistically superior to that of an initial model constructed of only 2 components (AR and PI) (F statistic = 24.1 and 64.0, respectively; P < 0.01 in both cases). In the case of data set 3, performance of the 3 and 2 component models were not significantly different (F statistic = 1.0, P = 0.314).

**Table 1:** Mean parameters and error values for the 3 component model.

	Data Set 1	Data Set 2	Data Set 3	Data Set 4
MAE	1.92 ± 0.23	2.13 ± 0.37	2.03 ± 0.13	1.11 ± 0.08
G_AR_	3.47 ± 0.15	2.71 ± 0.90	1.66 ± 0.26	1.27 ± 0.17
τ_AR_	1.30 ± 0.16	1.50 ± 0.26	1.58 ± 0.11	1.86 ± 0.08
G_PI_	0.10 ± 0.01	0.07 ± 0.04	0.06 ± 0.02	0.02 ± 0.01
G_PR_	0.005 ± 0.001	0.014 ± 0.006	0.006 ± 0.005	0.004 ± 0.001

Values represent the mean ± standard error of the mean. MAE; Mean absolute error, in mmHg. G_AR_, gain of the acute reversible component; G_PI_, gain of the progressive irreversible component; G_PR_, gain of the progressive reversible component; τ_AR_, time constant of the acute reversible component.

#### Higher order models

In subsequent studies, we found that further improvement of the model's dynamic performance required the addition of one or more high order functions of relatively arbitrary structure. Such additions greatly increased model complexity and arbitrariness while providing little additional insight or reduction in overall error, the MAE values already being quite modest (Table 1). Therefore, we considered the 3 component model to have achieved the study's objective with an appropriate balance between performance and complexity, and further elaborations of model structure were not pursued.

### Model performance for data from hybrid rats (data set 4)

The 3 component model performed well when applied to data from hybrid rats (data set 4), the performance being significantly better than that of an initial model having only 2 components (F statistic = 10.8; P < 0.01), and with the mean error value being well less than 2 mmHg (Table 1). G_AR_, G_PI_, and G_PR _values were reduced below the levels obtained for Dahl-S rats in data set 1-3 (Table 1), consistent with the observed attenuation of salt sensitivity of BP in Dahl strains in which the genetic determinants of their salt sensitivity have been diluted by cross breeding [13,25].

## Discussion

In the 1960s, development of the Dahl-S rat strain was instrumental in establishing the importance of genetic-environmental interactions in setting the long-term BP level. Today, descendents of this strain continue to be widely used to study the detailed mechanisms underlying salt-induced hypertension [26-29]. While many individual mechanisms have been studied, the progression of salt-induced hypertension has generally been discussed as if it were the result of a single (albeit complex) process or cascade. This may in part be due to the absence of suitably detailed time course data, which has become available only recently following the development of BP telemetry. In the present study we have demonstrated that the main dynamics of salt-induced hypertension in Dahl-S rats can be well represented as a multi-component process consisting of three major elements that differ in several fundamental characteristics, such as their time course and reversibility. Because each phase or component may involve a unique set of underlying mechanisms or genetic determinants, taking this multi-component nature in to account may have important ramifications for understanding and investigating the causes of salt-induced hypertension in this experimental model, and possibly others.

### Model components

**Figure 4:**
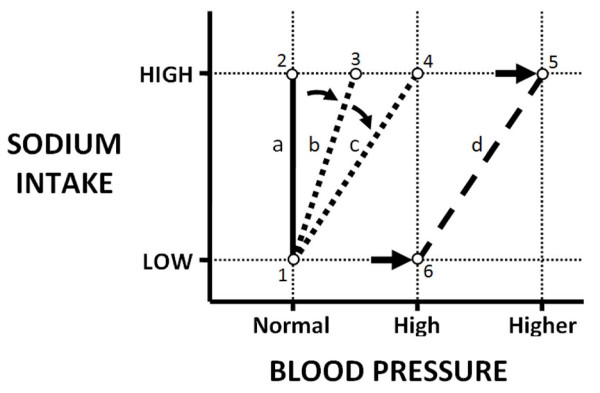
**Features of the chronic pressure natriuresis relationship**. Line-a corresponds to the steady state relationship between salt intake and BP for a salt resistant individual in which changes from low to high salt intake is not associated with a change in steady state BP (points 1 vs. 2). Acute salt sensitivity of BP (Acute-Reversible component of the model) corresponds with a reduced slope of this relationship (e.g. line-b), such that increases in salt intake leads to increases in steady state BP (points 1 vs. 3), and restoration of a low salt intake will return BP along line-b to the original level (point 1). Dahl-S rats exhibit acute salt sensitivity which appears to progressively worsen with salt exposure (Acute-Reversible component of the model, line c). The Progressive-Irreversible component of the model is represented by a progressive salt-induced rightward shift of the relationship along the x-axis to high BP levels (line d). The irreversible nature of the shift is associated with a shift in the baseline (from point 1 to 6), such that BP will remain elevated even following a return to low salt intake along line-d. The three component model is able to represent any salt-induced increase in BP (e.g. from the initial baseline, point 1, to a salt-induced increase in BP, point 3, 4, or 5) in terms of the initial slope and position of the relationship at baseline, combined with the independent effects of salt intake on the slope and position of the relationship.

Theoretical analyses of BP regulation have pointed to the fundamental role of the steady state relationship between salt intake and BP (the "chronic pressure natriuresis relationship" or "renal function curve", Figure 4) in setting the long-term BP level [3,8]. The slope of this relationship has been used to define the acute salt sensitivity of BP in previous analyses [3,8]. In our current model, we used three components to better represent the evolving steady state relationship between salt intake and BP during the development of salt-induced hypertension. As is evident in Figure 4 and explained below, the combination of these three components is capable of representing essentially any salt-induced change in the steady state level of BP.

#### First component (Acute-Reversible)

**Figure 5:**
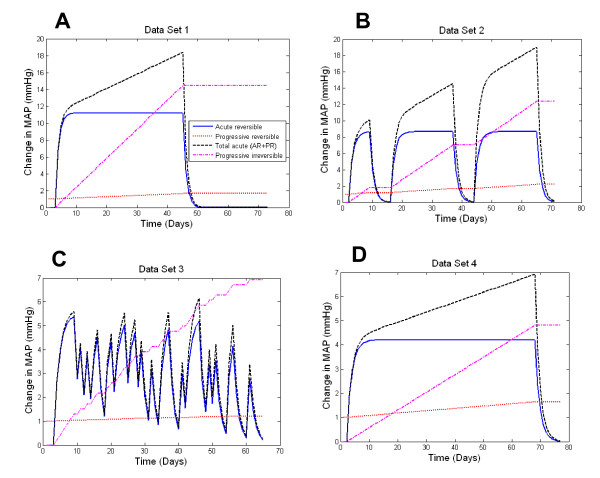
**Contribution of individual model components to the overall behavior of the 3 component model**. In addition to the three components of the model (Acute-Reversible, Progressive-Reversible, Progressive-Irreversible), the net effect of the interaction between the Acute-Reversible and Progressive-Reversible components (the total amount of salt-induced hypertension that can be reversed by salt restriction) is also plotted ("Total acute (AR+PR)"). The contributions are shown separately for data set 1 (A), data set 2 (B), data set 3 (C), and data set 4 (D).

In salt-sensitive subjects, salt loading leads to corresponding changes in BP within days to weeks, coinciding approximately with the time course of the reestablishment of salt balance. In our model, this phenomenon was accounted for by a linear transfer function, a simple reversible process that exhibited a time constant of one to two days (Table 1). Such time constant values correspond reasonably well with the time course of the initial phase of salt induced BP change in rats [22] (Figure 1). The gain of this component (G_AR_) represents the degree of acute salt sensitivity of BP and is inversely proportional to the slope of the chronic pressure natriuresis relationship [3,8]. As is evident in Figure 5, this component provided an important contribution to the overall behaviour of the 3 component model.

#### Second component (Progressive-Irreversible)

In addition to acute salt-sensitivity of BP, high salt intake has also been associated with a progressive rise in BP over a period of many months in regular Sprague Dawley rats [9,24] and chimpanzees [10] and decades in humans [11,12]. Lewis Dahl selectively bred Sprague Dawley rats for this characteristic, leading to the creation of the Dahl salt-sensitive rat strain in which this response to salt was greatly accelerated and amplified [30]. In a recent study, we found that this progressive effect of salt on BP appeared to be distinct from the initial acute response to salt loading in Dahl-S rats [13]. The data produced in these studies (data sets 1, 2, and 4) also illustrated that the slow and progressive phase of salt induced hypertension in this model was not fully reversible, a feature that Dahl had referred to as a self-sustaining characteristic of salt induced hypertension [15].

The 2^nd ^component (Progressive-Irreversible) of our model consisted of a simple integrator that imparted a cumulative and fully irreversible BP response to increased salt intake. Once developed, this component would provide a sustained elevation of BP irrespective of subsequent salt intake. This form of irreversible elevation of BP corresponds with a rightward shift of the chronic pressure natriuresis relationship to higher BP levels (Figure 4), a shift that has been shown to accompany the progressive and not-fully reversible salt-induced elevation of BP in the Dahl-S rat [13]. While this 2^nd ^component appeared to contribute importantly to the overall model behaviour (Figure 5), an initial model consisting only of these 2 components remained limited in its dynamic performance. In an effort to improve model performance, we added a 3^rd ^component which was capable of representing a reversible form of progressive salt induced hypertension [13] (see below) and also allowed for a more complete representation of changes in the chronic pressure natriuresis relationship.

#### Third component (Progressive-Reversible)

**Figure 6:**
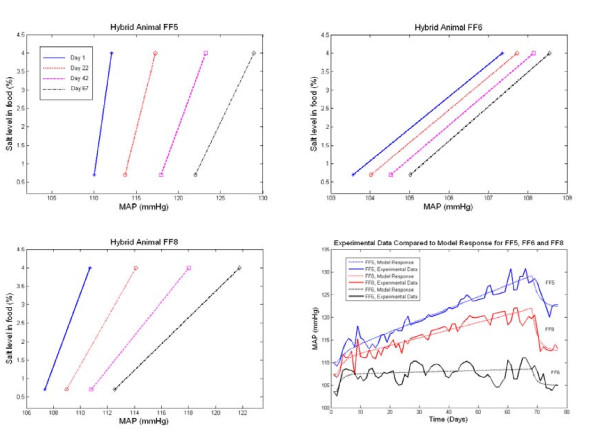
**Calculated changes in the chronic pressure natriuresis relationship associated with model simulations of the development of salt induced hypertension**. The graphs illustrate calculated changes in the chronic pressure natriuresis relationship for three individual hybrid rats of data set 4 (Rats FF5, FF6, and FF8). The relationship is plotted for days 1, 22, 42, and 67 of the protocol. The original BP time course data and corresponding model output for each of the three rats are shown in the fourth panel.

Like the 2^nd ^component of our model (above), the 3^rd ^component was also capable of representing a slow and progressive development of salt-induced hypertension. However, in contrast to the irreversible character of the 2^nd ^component, the progressive effects of the 3^rd ^component could be rapidly reversed by restoration of a normal salt intake. This model behaviour was implemented by imposing an effect of high salt intake to cause a progressive increase in acute salt-sensitivity of BP (Figure 3). This aspect of the 3^rd ^component is important, as it can account for a number of features evident in the data sets used in this study, including the manner in which the BP response to salt restriction increases in amplitude with increasing duration of exposure to high salt (Figure 1b). This effect amounts to a progressive salt-induced worsening of acute salt-sensitivity and corresponds with an effect of chronic high salt intake to reduce the slope of the chronic pressure natriuresis relationship (Figure 4) [13,14]. This progressive-reversible component appeared to provide an important contribution to model behaviour, particularly in data sets 1, 2, and 4, in which it allowed for a progressive rise in BP that was partly reversible (Figures 1 and 5). Its role in individual rats from data set 4 is illustrated in Figure 6. While this form of salt-induced salt sensitivity has not been widely considered in the past, it is evident in data from chimpanzees in which 22 months of salt loading was associated with a progressive rise in BP that was nevertheless reversible upon restoration of normal salt intake [10]. Such a process is also consistent with the age-associated increases in acute salt-sensitivity in humans [31,32]. While slow increases in acute salt-sensitivity in humans with age have been attributed to ageing itself, it is equally plausible that they are the result of an increasing period of exposure to a high salt intake, since a salt intake is typically high throughout life in Western societies [4].

We are not aware of a specific mechanism which could account for a salt-induced worsening of acute salt-sensitivity. However, in the Dahl-S rat, the development of salt-induced hypertension is accompanied by progressive changes in renal structure and an accumulation of renal lesions [33-35]. Such changes could potentially underlie a worsening of salt-sensitivity, similar to the suggested linkage between subtle changes in renal structure and the development of salt sensitivity in other experimental models [36].

### Strengths and limitations

The main purpose of our study was to investigate the time course of the development of salt-induced hypertension in Dahl-S rats and to determine if it could be well represented as a system with multiple components. In this regard our model has performed well, as it was able to provide a reasonable representation of the steady state components of previously published time course data for the development of salt-induced hypertension in the Dahl rat strain. This result is consistent with the hypothesis that multiple distinct phases or components are involved in the development of this form of hypertension.

An important strength of the model is its comprehensive ability to represent changes in the chronic pressure natriuresis relationship associated with the development and progression of salt-induced hypertension. By comprehensive, we refer to the ability of the model to represent virtually any salt-induced increase in the baseline BP level in terms of the initial slope of the chronic pressure natriuresis relationship and any subsequent salt-induced change in the position and/or slope of the relationship (Figure 4). In addition, the model may be useful to compare the contribution of individual components to the dynamics of salt-induced hypertension between individuals, and potentially between different strains or species. Figure 6, for example, illustrates the role of each of the three model components in individual hybrid rats of data set 4. Being second generation hybrids (F2 progeny of a cross of Dahl salt sensitive and Dahl salt resistant rats), these rats exhibit wide inter-individual variations in the characteristics of their salt-induced BP changes [13] (Figure 6). Nevertheless, the 3 component model was well able to represent the changes in BP and the corresponding changes in the chronic pressure natriuresis relationship.

A limitation of our model is that, while it was able to represent the salt-induced changes in steady state BP, it was not able to follow the most rapid dynamics exhibited by the BP data. This was particularly evident in the case of data set 3, a data set dominated by frequent changes in salt intake. In the case of data sets 1, 2, and 4, this limitation was also apparent as an inability to fully reproduce the BP transient associated with a sudden return of salt intake to control levels. Thus, while the performance of our model suggest that the time course of salt-induced hypertension can be approximated by a 3 component process, we must acknowledge that additional complexities exist, particularly with respect to the most rapid dynamics contained within the data.

A second limitation of our model is that it links dietary salt levels to BP responses without accounting for specific intermediary processes or mechanisms. While our model structures were based on time course data rather than specific mechanisms, they nevertheless do inform us about the arrangement and complexity of mechanisms underlying the development of salt-induced hypertension. Most importantly, our results suggest that salt-induced hypertension is not simply the result of one single process. On the contrary, it would appear to be the result of a multi-component process, with each phase or component differing with respect to features of timing, reversibility, and representation in the chronic pressure natriuresis relationship. From a mechanistic point of view, this is an important insight, since this implies that each individual component of our model may have a unique set of underlying mechanisms and genetic determinants.

## Conclusion

The effect of salt on BP is often presumed to be the result of a single process or cascade. Our model results demonstrate that the progression of salt-induced hypertension in the Dahl-S salt-sensitive rat can be well represented as a multi-component process, with each component having distinct characteristics of timing and reversibility. In addition, each component also has a corresponding distinct action on the slope or position of the chronic pressure natriuresis relationship. As we have shown here, this relationship not only dictates the steady state level of BP occurring for a given level of salt intake, but can also be used to describe the development of salt-induced hypertension in the Dahl-S rat model. An important implication of these findings is that each of the 3 components of our model may correspond with a unique set of underlying mechanisms and/or genetic determinants. Such components may therefore be important to consider in any comprehensive explanation of the etiology of salt-induced hypertension. In future studies of the Dahl rat or other salt-sensitive models, the mechanisms or characteristics associated with each individual component could be addressed through modest additions to standard experimental protocols that would permit the acute and progressive phases of salt induced BP changes, and their degree of reversibility, to be distinguished or quantified.

## Authors' contributions

VIM was responsible for implementing model structures in software and assessing and documenting model behaviour. JVR was responsible for overseeing model implementation and evaluation. BVV initially conceived of the study, coordinated manuscript preparation, and provided original data used in model development and evaluation. All three authors contributed to the design of the study and development of model structures. All authors read and approved the final manuscript.
